# Supplementary material for: Association of Combined Sero-Positivity to Helicobacter pylori and Streptococcus gallolyticus with Risk of Colorectal Cancer
Source: Microorganisms. 2020 Oct 30;8(11):1698. doi: 10.3390/microorganisms8111698 (PMC7693002; doi:10.3390/microorganisms8111698)
Supplement: Supplementary file 1 [file microorganisms-08-01698-s001.pdf]

Table S1: SGG Gallo2178 and HP VacA combined status and colorectal cancer risk, by time between blood draw and diagnosis.

	Controls n (%)	Cases n (%)	OR (95% CI)[a]	Among matched pairs with BMI and education available Controls n (%)	Cases n (%)	OR (95% CI)[a]	OR (95% CI)[b]
**Overall**							
HP VacA-/SGG Gallo2178-	2640 (65)	2563 (63)	1.00 (Ref)	2191 (65)	2128 (63)	1.00 (ref)	1.00 (ref)
HP VacA+/SGG Gallo2178-	1260 (31)	1303 (32)	1.08 (0.98, 1.19)	1061 (31)	1091 (32)	1.07 (0.96, 1.20)	1.06 (0.95, 1.19)
HP VacA-/SGG Gallo2178+	73 (2)	66 (2)	0.93 (0.66, 1.31)	67 (2)	58 (32)	0.89 (0.61, 1.28)	0.87 (0.60, 1.25)
HP VacA+/SGG Gallo2178+	90 (2)	131 (3)	**1.54 (1.16, 2.04)**	78 (2)	120 (4)	**1.63 (1.21, 2.20)**	**1.57 (1.16, 2.12)**
**<10 years between blood draw and dx**							
HP VacA-/SGG Gallo2178-	1774 (64)	1706 (62)	1.00 (Ref)	1629 (64)	1583 (62)	1.00 (ref)	1.00 (ref)
HP VacA+/SGG Gallo2178-	875 (32)	900 (33)	1.09 (0.96, 1.22)	808 (32)	816 (32)	1.05 (0.93, 1.19)	1.04 (0.92, 1.18)
HP VacA-/SGG Gallo2178+	47 (2)	56 (2)	1.25 (0.83, 1.87)	46 (2)	51 (2)	1.14 (0.75, 1.74)	1.13 (0.74, 1.72)
HP VacA+/SGG Gallo2178+	70 (3)	104 (4)	**1.58 (1.15, 2.18)**	65 (3)	98 (4)	**1.58 (1.14, 2.19)**	**1.53 (1.10, 2.12)**
**≥10 years between blood draw and dx**							
HP VacA-/SGG Gallo2178-	865 (67)	856 (66)	1.00 (Ref)	561 (66)	544 (64)	1.00 (ref)	1.00 (ref)
HP VacA+/SGG Gallo2178-	385 (30)	403 (31)	1.08 (0.90, 1.29)	253 (30)	275 (32)	1.15 (0.92, 1.44)	1.15 (0.92, 1.44)
HP VacA-/SGG Gallo2178+	26 (2)	10 (1)	**0.40 (0.19, 0.82)**	21 (2)	7 (1)	**0.35 (0.15, 0.84)**	**0.34 (0.14, 0.81)**
HP VacA+/SGG Gallo2178+	20 (2)	27 (2)	1.37 (0.74, 2.52)	13 (2)	22 (3)	1.80 (0.85, 3.78)	1.64 (0.78, 3.47)

[a]Conditional logistic regression model (controls matched to cases on sex, self-reported race/ethnicity, date of birth, and date of blood collection); [b] Conditional logistic regression model with adjustment for education (≤HS or GED, >HS - <college graduate, ≥college graduate), and BMI (<25, 25- <30, ≥30 kg/m^2).
